# A Latent Profile Analysis of Precarity and Its Associated Outcomes: The Haves and the Have-Nots

**DOI:** 10.3390/ijerph19137582

**Published:** 2022-06-21

**Authors:** Andrea Bazzoli, Tahira M. Probst, Jasmina Tomas

**Affiliations:** 1 Department of Psychology, Washington State University Vancouver, Vancouver, WA 98686, USA; probst@wsu.edu; 2 Department of Psychology, University of Zagreb, 10000 Zagreb, Croatia; jtomas@ffzg.hr

**Keywords:** precarity, economic stressors, latent profile analysis, economic insecurity, precariat

## Abstract

A continuing debate on the nature of precarity surrounds its defining characteristics and identification of what constitutes precarity. While early sociological work argued that people either experience precarity or they do not (i.e., the haves and the have-nots), subsequent researchers have gone to great lengths to argue for a more nuanced approach with multiple distinct classes of precarity. Using cross-lagged data from *n* = 315 U.S. employees collected during the COVID-19 pandemic, we took a person-centered approach to address this central question and uncover latent subpopulations of precarity. Specifically, we conducted a latent profile analysis of precarity using various objective and subjective indicators including perceptions of job insecurity, financial insecurity, prior unemployment experiences, per capita household income, skill-based underemployment, and time-based underemployment. While we anticipated different profiles based on income- vs. employment-based sources of precarity, the best-fitting solution surprisingly comported with Standing’s proposed two-class model. Moreover, membership in the precarious profile was associated with consistently more adverse subsequent outcomes across work, health, and life domains adding to the validity of the obtained two-profile structure. We discuss these results in light of potential loss spirals that can co-occur with the experience of precarity.

## 1. Introduction

Nearly 25 years ago, Cappelli [1] argued that “the old employment system of secure, lifetime jobs with predictable advancement and stable pay is dead (p. 17).” Such a statement presaged that the careers of employees would be characterized by increasingly precarious employment and punctuated by periods of unemployment, underemployment, financial insecurity, and job insecurity. Indeed, as Cappelli foresaw, while the traditional 9-to-5 permanent job with a single employer remains the dominant mode of employment in industrialized countries [2,3], it is increasingly being replaced and supplemented by more precarious forms of employment arrangements, including freelancing, independent contracting, casual work, online platform work, on-call workers, zero-hour contracts, and gig work. Indeed, research from the EU-27 [4] found increasing use of nine unique non-standard forms of employment, including platform work, casual work, and voucher-based work. Estimates from the United States [5] suggest that approximately 36% of workers fall into these categories and this percentage is expected to continue to rise with 80% of large organizations reporting [6] they are planning to decrease their reliance on traditional employees and increase their contingent workforce. At the same time as these workforce changes are occurring, decades of research on the economic stressors (i.e., unemployment, underemployment, financial insecurity, and job insecurity) that often accompany such precarious work has proliferated, with meta-analytic findings [7,8,9,10] demonstrating consistent and adverse effects on employees, organizations, and society in general.

A continuing debate on the nature of precarity surrounds its defining characteristics and identification of what constitutes precarity. While early sociological work (e.g., [11]) argued that people either experience precarity or they do not (i.e., the haves and the have-nots), others have gone to great lengths to argue for a more nuanced approach with multiple distinct classes and forms of precarity (e.g., [12]). Using a latent profile analysis approach and cross-lagged data from U.S. employees collected during the COVID-19 pandemic, we seek to address this central question by investigating and uncovering latent subpopulations of precarity.

## 2. Literature Review and Theory-Based Expectations

### 2.1. Definition of Precarious Employment

Precarious employment has traditionally been defined by what it is not, namely, the standard employment relationship [13] consisting of a full-time secure job often accompanied by benefits such as paid leave and retirement contributions. Thus, precarious employment is often characterized by the objective nature of the contractual employment relationship including work that is temporary, insecure, part-time, on-call, seasonal, and/or self-employed. However, in this paper, we argue that precarity encompasses both objectively defined features of the contractual relationship as well as subjectively held perceptions regarding those relationships. Moreover, precarity can occur due to deficits along the income and employment dimensions. Thus, we begin our literature review by developing our definition of precarity. Next, using that as a backdrop, we take a person-centered approach to better understand the nature of employee experiences of precarity and its outcomes. Finally, using a latent profile analysis approach, we empirically uncover profiles of precarity and their correlates.

Precarity has been defined as “a state of persistent insecurity with regard to employment or income” [14] and indeed, this dictionary definition comports with the economic stress framework initially developed by Voydanoff [15] and later refined by Probst [16]. In that framework, potential sources of economic stress include objective and subjective aspects of the income and employment dimensions of the worker/earner role. Employment-based sources of stress might include periods of employment instability characterized by unemployment spells, time- or skill-based underemployment, as well as concerns regarding future job loss (i.e., job insecurity). On the income side, economic stress might stem from economic deprivation, a loss of income or financial resources, an inability to meet one’s financial obligations, as well as more subjective perceptions of financial inadequacy, concerns, or worries. In this article, we argue that this economic stress framework can be used to explore possible latent profiles of precarity by examining the extent and nature of the co-occurrence of different forms of employment- and income-based sources of insecurity. In doing so, we hope to contribute to this IJERPH special issue on precarious employment by better understanding the nature of precarity and its psychosocial consequences.

### 2.2. Issues in Measuring Precarity

Measuring precarity is not as straightforward as measuring other organizational constructs due to the difficulty of obtaining a satisfactory operationalization and its large variability across institutional and cultural contexts [17,18]. Scholars have attempted to measure precarity in three main ways: first, it has been advanced that employees’ precarity can be derived by their objective contractual arrangements, thus equating precarity with temporary or nonstandard contracts. Following this line of reasoning, workers on a fixed-term contract are considered precarious [19]. Second, it has been proposed to combine objective (i.e., contractual arrangements) and subjective (e.g., perceived job insecurity) characteristics, but it remains unclear how these are combined, and indeed they have been analyzed separately in most instances see e.g., [18]. Third, a recent strand of literature began analyzing economic vulnerability using latent class analysis [20]: in this context, and throughout this contribution, classes are discrete latent variables that represent subgroups in the sample. This approach can be useful in understanding precarity because it can accommodate several indicators of latent precarity classes, it takes into account measurement error, it can be easily implemented with most statistical software, and it hinges on local independence (i.e., latent variables explain why observed indicators are related to one another). Given the clear mathematical and substantive advantages, we use latent classes to understand the nature of precarity and base this approach on a well-established framework of income- and employment-based sources of economic stress.

### 2.3. A Person-Based Approach to Understanding Precarity

We consider six distinct objective and subjective economic stressors that can be seen as observed indicators of precarity, namely: job insecurity, financial insecurity, prior unemployment experiences, per capita household income, skill-based underemployment, and time-based underemployment.

#### 2.3.1. Employment-Based Sources of Economic Stress

The most prevalent (and researched) sources of employment-based economic stress include unemployment, underemployment, and job insecurity. Unemployment occurs when a person is actively searching for employment but is unable to obtain work. Episodes of unemployment can be objectively measured by assessing the quantity and duration of periods that individuals have experienced unemployment. Meta-analyses indicate that unemployment is one of the most significant stressors an individual can face and has been associated with higher incidence of all-cause mortality [21], suicide [22], depression [23], and even a loss of cognitive ability [24]. Underemployment occurs when a worker is employed in a job that is below their full capacity [8]. This can be due to a mismatch in their desired vs. obtained work hours (time-based unemployment; e.g., part-time worker desiring a full-time position) or to being overqualified (skills-based underemployment; e.g., a worker who has more experience, education, or skills than required for their current position). Systematic reviews of the literature [8] indicate that underemployment is related to job attitudes, performance, turnover intentions, and psychological well-being. Job insecurity is a subjective perception reflecting worker concerns regarding the perceived stability and continuance of their employment [25,26]. A recent meta-analysis [7] documented the adverse effects of job insecurity on several outcomes, including work-related outcomes such as job satisfaction, organizational commitment, engagement, burnout, turnover, safety, and job performance, as well as individual outcomes including life satisfaction, mental and physical health, depression, anxiety, and work-life conflict.

#### 2.3.2. Income-Based Sources of Economic Stress

As with employment-based sources of economic stress, income-based sources can be objectively measured and subjectively assessed. For example, per capita household income can be used as an indicator of economic deprivation and has been found in meta-analytic research [27] to be associated with various measures of subjective well-being, including life satisfaction, happiness, and perceived quality of life. Financial insecurity, on the other hand, is a subjective evaluation of one’s current financial state and involves concerns or worries that one’s finances are inadequate for meeting one’s current financial needs and obligations [16]. Such financial stress has been found to predict workplace injuries and accident underreporting [28], as well as depressive symptomatology and self-reported anxiety [29].

### 2.4. Theory-Based Expectations of Latent Profiles and Their Outcomes

Because latent profile analysis is by definition an exploratory technique [30] that attempts to uncover meaningful groups from observed data, we do not pose specific research hypotheses regarding the specific number or nature of the resulting profiles. However, below we present theory-based expectations for two possible different emergent latent profiles, i.e., emergence of multiple precariat profiles vs. emergence of a single “precariat”.

#### 2.4.1. Multiple Profiles Argument

Based upon the previously reviewed model of economic stress, we can develop possible profiles of precarity that might emerge when assessing numerous sources of economic stress, including unemployment experiences, time- and skills-based underemployment, job insecurity, per capita household income, and financial insecurity. Specifically, the typology of income- versus employment-based sources of economic stress suggest that the following distinct profiles of precarity might emerge:

- High income-based precarity and high-employment-based precarity
- Low income-based precarity and low-employment-based precarity
- High income-based precarity but low-employment-based precarity
- Low income-based precarity but high-employment-based precarity

The first two profiles would be predicted by Conservation of Resources theory [31], which suggests that resources travel in caravans, such that resource gains beget further gains, but also loss spirals occur such that individuals exposed to prior resource loss (e.g., unemployment, underemployment, etc.) are at greater risk of future resource loss. Thus, groups with greater income precarity might be more at risk of employment-based precarity (and vice versa), whereas individuals with few income-based sources of stress might be less at risk of experiencing employment-based sources of economic stress (and vice versa).

On the other hand, research on the important distinction between latent and manifest benefits of employment might support the additional emergence of the latter two profiles. Jahoda’s latent deprivation model [32] proposes that employment offers significant latent benefits, including time structure, activity, social contact, collective purpose, and status) that are distinct from the manifest benefits of one’s job as an income source. Whereas Jahoda argues that the loss of these latent benefits are the primary cause for adverse outcomes of unemployment, Fryer’s agency restriction theory proposes that it is the loss (or threat of loss) of the manifest benefits of income that are of greater importance in predicting the adverse effects of unemployment (and, by extension, threats to one’s employment via job insecurity or underemployment). Given this important distinction between latent vs. manifest benefits of employment (and research indicating they are only modestly correlated [33]), one might expect that profiles could emerge whereby individuals are experiencing primarily income-based precarity (but not necessarily employment-based precarity) and vice versa.

#### 2.4.2. The Single “Precariat” Argument

In contrast to the multiple profile arguments, scholars e.g., [11,34,35] have argued that the precariat (i.e., a class that represents employees that are exploited by means of “flexible” contracts, weak labor protections, no occupational identity, and vulnerability to employer disciplinary measures) is best conceptualized as a homogenous class. Standing [11] noted that precarious employees experience both income- and employment-based precarity, which he labels “relations of distribution” and “relations to production,” respectively. In addition to this, labor law traditionally grants considerably less rights to precarious employees (i.e., “relations to the state”) In other words, the single precarity class would consist of people living through insecure jobs interspersed with unemployment spells and with unstable access to housing and other public resources. Further, precarious employees are predicted to have a lower income that comes almost exclusively from wages, that is, they lack non-wage perks such as paid time off, medical and family leave allowances, health insurance, company-sponsored retirement options. In sum, employment instability, income vulnerability, and lack of labor protections are all necessary conditions for the precarity class.

Similarly, anthropological research carried out of North America and Europe [34,35] has long recognized the existence of two classes of workers: those who enjoy a permanent and salaried job and those who do not. This would suggest the presence of only two latent classes: the haves (i.e., economically secure individuals along employment and income dimensions) and the have-nots (i.e., the precariat).

#### 2.4.3. Outcomes of the Latent Profiles

Finally, we expect that membership in distinct profiles of precarity will have differential associations with known outcomes of economic stressors documented in previous research [7,8,9] in three areas: employees’ lives outside of work (i.e., physical health, life satisfaction), the interface between work and family (i.e., work-family conflict), and organizational outcomes (i.e., job satisfaction, affective commitment, task performance, psychological contract breach). Specifically, based on that extensive literature reviewed earlier regarding the outcomes of income- and employment-based sources of economic stress, we hypothesize that membership in profiles characterized by greater precarity (e.g., high income and high employment-based precarity) will be associated with more negative standing on these variables compared to membership in profiles reflecting less precarity.

## 3. Materials and Methods

### 3.1. Participants and Procedure

Data were collected in October 2020 and December 2020 as a part of a larger project examining the impact of COVID-19 on work- and life-related outcomes [20,26,36,37,38]. U.S.-based Mechanical Turk (MTurk) was used to collect a convenience sample of employees who (a) worked at least 20 h per week outside of this platform, (b) had a supervisor, and (c) worked for the same employer throughout the project. To increase data validity, data were collected only from “high reputation” MTurk respondents who previously completed at least 100 tasks coupled with an approval rating of 90% or higher [39]. Furthermore, data from employees flagged as careless respondents or multivariate outliers [40] were excluded.

Our sample consisted of 315 respondents who competed both waves and lived in 45 states plus the District of Columbia. Most participants were male (i.e., 59%, whereas 41% self-identified as female), identified as White (i.e., 76%, whereas 10% identified as Asian, and 10% belonged to other racial minorities), and were 39.90 years old on average (SD = 10.52). Almost half of the sample held a college degree (47%). The industries that were most represented in the sample were healthcare (19%), manufacturing (12%), finance (12%), and entertainment (12%). Respondents worked on average 39.45 h per week (SD = 8.37).

### 3.2. Measures

A two-month interval lagged design was utilized to reduce common method bias that could inflate covariances between study variables [41]. Indicators of precarity (job insecurity, financial inadequacy, skill- and time-based unemployment, previous unemployment spells, and income) were measured in October 2020 (Time 1), whereas outcomes of interest (physical health, life satisfaction, work-family conflict, job satisfaction, affective commitment, and psychological contract breach) were measured in December 2020 (Time 2). Measures used in this study are presented in Table 1.

### 3.3. Data Analysis

We used Mplus 8.7 for all statistical analyses presented in this contribution with the aim of uncovering the optimal number of latent classes. Extant literature (see e.g., [20,46]) outlined several statistical and substantive criteria, but there is no definitive criterion to guide the enumeration procedure. As such, in addition to the substantial theoretical value of the extracted classes, analysts should consider the Lo-Mendell-Rubin likelihood ratio test (LMR) and classification accuracy (i.e., entropy) when selecting a solution. Both statistical indices have been shown to yield consistent results in simulations.

In mixture models, the empirical variance-covariance matrix is explained by a set of underlying latent categorical variables. However, analysts are often interested in estimating latent classes using only a subset of variables, instead of the entire matrix, and use the remaining variables as predictors or outcomes. This gives rise to the well-known auxiliary variable issue [47], that is, an undesirable shift in latent classes estimation if covariates and outcomes were included in the latent class estimation procedure concurrently. To this end, we use the method first introduced by Bolck, Croon, and Hagenaars [48], which can be seen as an ANOVA-like model: the outcome’s mean across latent classes is estimated, and equality of these means is later tested using a Wald’s chi square test.

## 4. Results

### 4.1. Preliminary Analyses

None of the scale items demonstrated skewness and kurtosis that exceeded 3.0 and 10.0, supporting approximate normality assumptions [49]. We estimated a confirmatory factor analytical model (excluding single-item measures), which fit the data well (χ^2^(362) = 546.46, CFI = 0.99, RMSEA = 0.04, SRMR = 0.05), confirming the goodness of our measurement model. Study variables, descriptive statistics, correlations, and internal consistency indices are presented in Table 2. As expected, all our indicators of precarity (i.e., job insecurity, financial inadequacy, skill- and time-based unemployment, previous unemployment spells, and income) correlated in the expected direction with the outcomes.

### 4.2. Latent Class Enumeration

Table 3 provides model fit and estimated class size for the three solutions that were tested. To retrieve the best fitting model, we used the following benchmarks: LMR test = significant (i.e., rejecting a solution with k-1 classes in favor of the k model), relative entropy = closest to 1, and substantial theoretical relevance of classes. All models showed a comparable and very high entropy, reflecting a high classification accuracy. However, the 4-class model encountered estimation issues (i.e., some standard errors could not be estimated), which might indicate that too many classes have been extracted. Corroborating that, the smallest class count based on most likely class membership is *n* = 6. This model is therefore excluded from further consideration. The LMR test further showed that a 2-class solution is statistically preferrable to a 3-class one. As was the case for the 4-class solution, the smallest class in the 3-class solution had a very low count (*n* = 7).

To ascertain that we had enough power to correctly reject a 3-class solution, and we were not simply rejecting a model with a substantively important but not highly prevalent class, we computed statistical power using Equation (7) and Table 4 in [50] and an effect size of 0.3. Results showed that *n* = 205 is needed to reach power = 0.80, showing that we had enough statistical power to correctly reject a 3-class model.

Further, the 2-class model had substantial theoretical relevance (e.g., [11]): class 1 represents workers that are more insecure, less financially stable, and overall more economically vulnerable (i.e., the have-nots) whereas class 2 represents workers that are more financially stable, more secure, and less vulnerable (i.e., the haves). For these reasons, we chose a 2-class solution for all further analyses. The top half of Table 3 shows the descriptive statistics from key study variables for participants assigned to either latent class based on their most likely class membership.

### 4.3. Outcomes’ Means across Latent Classes

The class-specific means for all the outcomes are presented in the bottom half of Table 4. As can be seen, compared to the Have-Nots, the Haves reported significantly higher life satisfaction (χ^2^(1) = 41.15; *p* < 0.001), physical health (χ^2^(1) = 36.61; *p* < 0.001), job satisfaction (χ^2^(1) = 34.98; *p* < 0.001), and affective commitment (χ^2^(1) = 21.94; *p* < 0.001). Additionally, they reported lower perceived contract breach (χ^2^(1) = 23.36; *p* < 0.001) and work-family conflict (χ^2^(1) = 17.76; *p* < 0.001).

## 5. Discussion

We estimated a latent class analysis model to extract several latent subpopulations of precarious employees. Results showed that the best-fitting solution is a 2-class solution, which we dubbed haves and have-nots. The former class showed lower levels of all precarity indicators, suggesting that employees in this class are economically secure along employment and income dimensions. In contrast, the latter class showed higher levels of all precarity indicators, indicating that this class likely represents precarity. We further investigated whether these two classes exhibited significantly different means with regard to outcomes across several domains of workers’ lives (i.e., general health, work and family, and organizational outcomes). Our findings showed that generally, the haves tend to exhibit better outcomes compared to the have-nots across all three domains investigated.

### 5.1. Implications for Theory

Despite the longstanding strand of research devoted to understanding precarity, researchers are still debating about the optimal approach to defining and measuring this phenomenon [18,51]. In response, this study is one of the few that encompasses a wide array of both objective and subjective, as well as income- and employment-based sources of insecurity to uncover latent subpopulations of precarious employees using a person-centered approach. A 2-class solution derived from latent profile analysis indicates that precarious employees form a homogeneous group in terms of exposure to various economic stressors. Relative to the economically secure haves, the have-nots represent a financially deprived, job insecure workforce occupying positions which are inferior by required skill levels and work hours, and whose employment record is intersected by more episodes of unemployment. As such, our findings are in line with Standing’s work [11] who used the term precariat to describe a social class of employees who not only struggle to make ends meet, but also struggle to overcome and alter their precarious situation. Following his work, causes of one’s precarity are multi-dimensional and intertwined including inadequate salary and access to other organizational resources (e.g., training, paid time off), employment instability, and lack of state and non-state social protection.

More recently, however, scholars have challenged the conceptual meaning of Standing’s precariat e.g., [51] mostly because it implies that a wide range of very heterogeneous people should be encompassed by only one distinct socio-economic group. Such classification from their perspective oversimplifies this complex social phenomenon and leads to the loss of its conceptual value. Notwithstanding the merits of these arguments, our results suggest that the definition of precarity should encompass both income- and employment-related (as well as subjective and objective) indicators, as delineated by the economic stress literature [15,16] and Conservation of Resources theory [52]. More specifically, membership in a precarious class can be explained by the pattern of scores that unequivocally delineate a vulnerable position in capitalist countries—precarious employees do not only experience economic stress related to inadequate income, but also work in suboptimal and insecure jobs which enable them little prospects of improving their precarious position in a society. As such, one source of insecurity (e.g., long and frequent episodes of unemployment) might spur exposure to other sources of insecurity (e.g., low income) and vice versa [52].

Additionally substantiating these conclusions, the second set of results demonstrates that precarious employees have more negative standing on a wide range of outcomes. Interpreted through the lens of COR theory, these findings hint at some of the mechanisms that potentially explain why changing one’s precarious position is not easy. Due to the lack of important resources (e.g., money, good employment), membership in a precarious class makes employees more vulnerable to future resource loss (e.g., health and good relationships with employer). Because initial loss begets future loss [31,52], precarious employees might more easily find themselves caught in a downward loss spiral. For example, insufficient income may deteriorate physical health which might make employees less fit for their jobs increasing in turn their job insecurity, which then further deteriorates employees’ health [53]. Such vicious cycles illustrate that precarious employees might easily find themselves confronted with chronic uncertainty in almost every sphere of their lives [11].

Finally, it should be noted that the latent subpopulations uncovered by the means of latent profile analysis –the haves and have-nots– cannot be entirely equated with the sociological term of social class. In this regard, our classification based on income and employment-related indicators of precarity most closely corresponds to the traditional model of class structures which define a person’s class through their relation to the means of production. Interestingly, the two classes in our sample are very similar with regards to their socio-demographic characteristics (i.e., gender, age, and race), with the only exception being that haves are more highly educated than have-nots. According to this approach, class is determined as a position referring to one’s hierarchical order in a societal division of labor and wealth [54]. However, more recent perspectives argue that the people in the same social class also share goals, identity, specific patterns of thinking, feeling, acting, and consumption patterns, [54,55]. Social class is therefore more than classification and the classification obtained in our study could serve as a basis for future refinements and explorations of more nuanced characteristics of haves and have nots.

### 5.2. Implications for Practice

The rapid pace of technological developments, increased reliance on artificial intelligence, algorithmic management of work and employment, the rise of the gig economy and the concomitant decline in the standard employment relationship means that researchers and practitioners alike need to better understand how these changes impact the precarity of employment and income among today’s workforce. The results of our study suggest that individuals in relatively secure positions and financial states (i.e., the haves) demonstrated significantly more positive outcomes on a host of variables; the “have nots” reported worse outcomes. This not only suggests a potentially widening gap between the haves and the have nots in society, but also spells trouble for employers and society at large.

Legally, there are important distinctions between what constitutes an “employee” versus a “worker.” Within the United States, an individual is considered an “employee” if they have been contractually hired for the benefit of a specific employer that has discretion over how and when the employee does their job and pays the employee wages along with any required benefits, taxes, or other forms of compensation. On the other hand, a worker is someone who has a non-employee relationship with a company (or customer), but rather is hired to do a specific task or duty. These individuals are typically non-salaried, file their own taxes, and are in control over how, where, and when they perform their negotiated duties.

As organizations increasingly shift their workforce to more precarious forms of employment (i.e., a shift to using “workers” rather than hiring “employees”), this may have the side-effect of a less healthy, productive, committed, and innovative workforce. Thus, from an organizational perspective, companies that depart from traditional notions of the standard employment relationship need to proactively consider how best to support the stability of income and employment among those workers (even if such workers are not “employees”). Similarly, from a societal and governmental perspective, this may call for a rethinking of current forms of state and non-state social protections as responses to the increasing precaritization of work and employment. For example, in the United States, there are some legal protections afforded to “employees” but far fewer granted to “workers.” As the labor force shifts to more precarious forms of employment, legislation and policy need to keep up with these shifts.

For example, prior to the COVID-19 pandemic, many precarious groups of workers (e.g., self-employed, gig workers) were ineligible for unemployment benefits. However, the CARES Act gave individual states the option of extending unemployment compensation to self-employed, independent contractors, gig-economy workers, and other individuals who would normally be ineligible for unemployment benefits if they had obtained a minimum level of income from self-employment in the prior year. In other words, to qualify, individuals did not need to provide proof of prior “employment”, but rather proof of prior “income” to substantiate their role in the labor force. Unfortunately, this special Pandemic Unemployment Assistance program ended in September of 2021, resulting in the loss of that critical safety net for those self-employed, freelance, gig, and/or part-time workers.

Just as income inequality has dramatically grown over the past several decades, the upcoming decades will likely see an equally dramatic shift in employment inequality in the labor force, in which the relatively fewer “have” employees who maintain the traditional standard employment relationship are granted access to better benefits and social safety nets, whereas the “have nots” who are employed in more precarious positions characterized by uncertain income and employment prospects see that precarity further magnified by the absence of eligibility for various social safety net programs.

### 5.3. Limitations and Directions for Future Research

Although this paper made several contributions to the precarity literature, some limitations should be acknowledged. First, we were not able to include employees’ contract type in our precarity indicators because the vast majority of workers in our sample were W-2 employees (i.e., they were considered employees by their organization, as opposed to independent contractors; see above). Future research should strive to include this as one of the indicators, as theory overwhelmingly suggests that it is one of the most relevant features of precarity [11,51], perhaps going beyond the traditional fixed-term vs. permanent dichotomy and including nonstandard working arrangements such as zero-hour contracts, and similar exploitative contracts. Other precarity indicators (e.g., qualitative job insecurity [56]) should be included in future analyses as well.

We were able to include precarity outcomes measured two months after the precarity indicators. Although this is valuable to establish temporal precedence, we treated precarity as a fixed (i.e., time invariant) characteristic of employees’ working lives. Future research should elucidate temporal mechanisms of precarity: analytical methods such as longitudinal latent profile analysis allow participants to move across classes (e.g., mover-stayer analyses) that have indicators at different occasions. Most interestingly, though, it is possible to specify predictors of class membership change in those analytical frameworks, allowing scholars to ask (and hopefully find answers to) questions related to what variables cause workers to enter or leave precarity. In turn, this could inform state programs aimed at improving precarious workers’ lives.

Lastly, our data were collected in the US, which are notorious for having labor laws that are heavily skewed towards employers: notably, in 26 out of 50 states employers can terminate employees at any time without just cause (provided that it is not illegal under federal or state law). It follows that all workers in these states are precarious in some way, as their contractual relationship might end without notice. We see values in replicating our findings in other national contexts with varying degrees of labor law protections and exploring the role of legal frameworks in shaping experiences of precarity.

## 6. Conclusions

Taking a person-centered perspective, this paper sought to uncover latent subpopulations of precarity. Our analyses showed that participants either experienced (i.e., have nots) or did not experience precarity (i.e., the haves), lending empirical evidence to some earlier sociological perspectives [11]. Further analyses also revealed that, compared to the haves, the have nots tend to show worse outcomes across work, health, and life domains.

## Figures and Tables

**Table 1:** Measures.

Construct	Reference	Response Range	Sample Item
Job Insecurity	[25]	0–2–3	“Unpredictable”
Financial Inadequacy	[28]	1–5	“I don’t have enough money to pay my bills.”
Time-based underemployment	Self-developed	1–5	“I am currently working fewer hours than I would like to” “I am earning less money compared to my previous jobs” “I am earning less money compared to other people in similar jobs”
Skill-based underemployment	Self-developed	1–5	“I have more education and training than is required for my current job” “I have more skills and experience than is required for my current job” “My current employment is a match for my educational background”
Previous unemployment	Self-developed	n/a	Number of unemployment spells (calculated as a sum of indicated unemployment instances experienced during the 5 years prior to the start of the pandemic and during the 6 months of the pandemic from April to October 2020)
Per capita income	Self-developed	1 (less than $10,000)–12 (more than $150,000)	Indicated total household income in 2019 divided by the number of people living in that household
Life satisfaction	[42]	1–7	“In most ways my life is close to my ideal.”
Physical health	Self-developed	1–7	“How would you rate your overall physical health?” (1-item measure)
Job satisfaction	Self-developed	1–7	“Overall, how satisfied are you with your current job?” (1-item measure)
Work-family conflict	[43]	1–7	“I have to miss life activities outside work due to the amount of time I must spend on work responsibilities.”
Affective commitment	[44]	1–7	“I would be happy to spend the rest of my career with this organization.”
Perceived contract breach	[45]	1–5	“I feel that my employer has come through in fulfilling the promises made to me when I was hired (reverse coded).”

**Table 2:** Descriptive statistics, correlations, and reliability.

	M	SD	1	2	3	4	5	6	7	8	9	10	11	12
1. Job Insecurity	0.76	1.11	(0.97)											
2. Financial Inadequacy	1.64	0.78	0.30 ***	(0.94)										
3. Time-Based Underemployment	2.27	0.91	0.32 ***	0.43 ***	(0.76)									
4. Skill-Based Underemployment	2.95	0.94	0.10	0.19 ***	0.28 ***	(0.71)								
5. Previous Unemployment	0.34	0.76	0.19 ***	0.36 ***	0.20 ***	0.11	---							
6. Per Capita Income	3.27	1.99	−0.07	−0.33 ***	−0.10	−0.07	−0.17 **	---						
7. Physical Health	3.99	0.79	−0.29 ***	−0.32 ***	−0.15 **	−0.10	−0.02	0.11	---					
8. Life Satisfaction	4.57	1.65	−0.37 ***	−0.31 ***	−0.22 ***	−0.24 ***	−0.15 **	−0.03	0.47 ***	(0.93)				
9. Work-Family Conflict	2.79	1.52	−0.31 ***	0.38 ***	0.26 ***	0.18 ***	0.12 *	−0.13 *	−0.29 ***	−0.33 ***	(0.83)			
10. Job Satisfaction	5.29	1.45	−0.46 ***	−0.21 ***	−0.28 ***	−0.20 ***	−0.16 **	−0.06	0.36 ***	0.59 ***	−0.33 ***	---		
11. Affective Commitment	4.66	1.73	−0.33 ***	−0.15 **	−0.27 ***	−0.21 ***	−0.21 ***	−0.03	0.25 ***	0.55 ***	−0.25 ***	0.76 ***	(0.96)	
12. Psychological Contract Breach	2.19	0.83	0.33 ***	0.26 ***	0.28 ***	0.24 ***	0.09	0.03	−0.26 ***	−0.45 ***	0.39 ***	−0.57 ***	−0.57 ***	(0.90)

Values on the diagonal (in parentheses) are Cronbach alpha coefficients. Coefficients for some variables could not be computed because either variables were measured using single-item measures (i.e., physical health and job satisfaction) or they measured objective characteristics (i.e., previous employment spells and per-capita income). * *p* < 0.05, ** *p* < 0.01, *** *p* < 0.001.

**Table 3:** Latent class models comparison.

Model	Class Size	Entropy	LMR LRT	*p*-Value
2 Classes	75% 25%	0.95	426.58	<0.001
3 Classes	76% 22% 2%	0.97	205.87	0.32
4 Classes	71% 19% 8% 2%	0.96	74.99	0.67

LMR LRT = Lo-Mendell-Rubin Likelihood Ratio Test.

**Table 4:** Latent classes descriptive statistics.

	Haves	Have-Nots
*Characteristics*		
Individuals in Class	241	74
Females	41%	42%
Males	59%	58%
Mean Age (SD)	40 (10.5)	40 (10.5)
White	75%	76%
Racial Minority	25%	24%
Education (Most Prevalent)	College (50%)	College (35%)
*Latent Class Indicators*		
Job Insecurity (SD)	0.15 (0.32)	2.64 (0.42)
Financial Inadequacy (SD)	1.47 (0.60)	2.01 (0.97)
Time-Based Underemployment (SD)	2.09 (0.78)	2.74 (1.13)
Skill-Based Underemployment (SD)	2.86 (0.94)	3.02 (0.92)
Previous Unemployment (SD)	0.24 (0.59)	0.65 (1.42)
Per Capita Income (SD)	3.32 (2.01)	0.65 (1.42)
*Outcomes*		
Physical Health (SE)	4.13 (0.05)	3.57 (0.11)
Life Satisfaction (SE)	4.96 (0.10)	3.55 (0.19)
Work-Family Conflict (SE)	2.52 (0.09)	3.51 (0.21)
Job Satisfaction (SE)	5.60 (0.08)	4.34 (0.19)
Affective Commitment (SE)	4.95 (0.11)	3.83 (0.21)
Psychological Contract Breach (SE)	2.05 (0.05)	2.63 (0.11)

## Data Availability

The data needed to evaluate the conclusions in this paper can be provided by the corresponding author upon reasonable request.
